# Supplementary material for: Resolving the full spectrum of human genome variation using Linked-Reads
Source: Genome Res. 2019 Apr;29(4):635–45. doi: 10.1101/gr.234443.118 (PMC6442396; doi:10.1101/gr.234443.118)
Supplement: Supplemental Material [file supp_gr.234443.118_Supplemental_longranger-2.2.zip › longranger-2.2.tar.bz2!/lr2.2-public/lib/stan/cmdstan/stan/doc/api/html/inv__chi__square_8hpp_source.html]

Stan: src/stan/prob/distributions/univariate/continuous/inv\_chi\_square.hpp Source File


|  |  |
| --- | --- |
|  | Stan  2.5.0  probability, sampling & optimization |


- Main Page
- Related Pages
- Namespaces
- Classes
- Files

- File List
- File Members

All Classes Namespaces Files Functions Variables Typedefs Enumerations Enumerator Friends Macros Pages

- src
- stan
- prob
- distributions
- univariate
- continuous

inv\_chi\_square.hpp

Go to the documentation of this file.

1 #ifndef STAN\_\_PROB\_\_DIST\_\_UNI\_\_CONTINUOUS\_\_INV\_CHI\_SQUARE\_HPP

2 #define STAN\_\_PROB\_\_DIST\_\_UNI\_\_CONTINUOUS\_\_INV\_CHI\_SQUARE\_HPP

3

4 #include <boost/random/chi\_squared\_distribution.hpp>

5 #include <boost/random/variate\_generator.hpp>

6

7 #include <stan/agrad/partials\_vari.hpp>

8 #include <stan/math/error\_handling.hpp>

9 #include <stan/math/constants.hpp>

10 #include <stan/math/functions/multiply\_log.hpp>

11 #include <stan/math/functions/value\_of.hpp>

12 #include <stan/meta/traits.hpp>

13 #include <stan/prob/constants.hpp>

14 #include <stan/prob/traits.hpp>

15 #include <stan/prob/internal\_math.hpp>

16

17 namespace stan {

18

19  namespace prob {

20

40  template <bool propto,

41  typename T\_y, typename T\_dof>

42  typename return\_type<T\_y,T\_dof>::type

43  inv\_chi\_square\_log(const T\_y& y, const T\_dof& nu) {

44  static const char\* function = "stan::prob::inv\_chi\_square\_log(%1%)";

45

46  // check if any vectors are zero length

47  if (!(stan::length(y)

48  && stan::length(nu)))

49  return 0.0;

50

51  using stan::math::check\_positive\_finite;

52  using stan::math::check\_not\_nan;

53  using stan::math::value\_of;

54  using stan::math::check\_consistent\_sizes;

55

56  double logp(0.0);

57  check\_positive\_finite(function, nu, "Degrees of freedom parameter",

58  &logp);

59  check\_not\_nan(function, y, "Random variable", &logp);

60  check\_consistent\_sizes(function,

61  y,nu,

62  "Random variable","Degrees of freedom parameter",

63  &logp);

64

65

66  // set up template expressions wrapping scalars into vector views

67  VectorView<const T\_y> y\_vec(y);

68  VectorView<const T\_dof> nu\_vec(nu);

69  size\_t N = max\_size(y, nu);

70

71  for (size\_t n = 0; n < length(y); n++)

72  if (value\_of(y\_vec[n]) <= 0)

73  return LOG\_ZERO;

74

75  using boost::math::digamma;

76  using boost::math::lgamma;

77  using stan::math::multiply\_log;

78

79  DoubleVectorView<include\_summand<propto,T\_y,T\_dof>::value,

80  is\_vector<T\_y>::value> log\_y(length(y));

81  for (size\_t i = 0; i < length(y); i++)

82  if (include\_summand<propto,T\_y,T\_dof>::value)

83  log\_y[i] = log(value\_of(y\_vec[i]));

84

85  DoubleVectorView<include\_summand<propto,T\_y>::value,

86  is\_vector<T\_y>::value> inv\_y(length(y));

87  for (size\_t i = 0; i < length(y); i++)

88  if (include\_summand<propto,T\_y>::value)

89  inv\_y[i] = 1.0 / value\_of(y\_vec[i]);

90

91  DoubleVectorView<include\_summand<propto,T\_dof>::value,

92  is\_vector<T\_dof>::value> lgamma\_half\_nu(length(nu));

93  DoubleVectorView<!is\_constant\_struct<T\_dof>::value,

94  is\_vector<T\_dof>::value> digamma\_half\_nu\_over\_two(length(nu));

95  for (size\_t i = 0; i < length(nu); i++) {

96  double half\_nu = 0.5 \* value\_of(nu\_vec[i]);

97  if (include\_summand<propto,T\_dof>::value)

98  lgamma\_half\_nu[i] = lgamma(half\_nu);

99  if (!is\_constant\_struct<T\_dof>::value)

100  digamma\_half\_nu\_over\_two[i] = digamma(half\_nu) \* 0.5;

101  }

102

103  agrad::OperandsAndPartials<T\_y, T\_dof> operands\_and\_partials(y, nu);

104  for (size\_t n = 0; n < N; n++) {

105  const double nu\_dbl = value\_of(nu\_vec[n]);

106  const double half\_nu = 0.5 \* nu\_dbl;

107

108  if (include\_summand<propto,T\_dof>::value)

109  logp += nu\_dbl \* NEG\_LOG\_TWO\_OVER\_TWO - lgamma\_half\_nu[n];

110  if (include\_summand<propto,T\_y,T\_dof>::value)

111  logp -= (half\_nu+1.0) \* log\_y[n];

112  if (include\_summand<propto,T\_y>::value)

113  logp -= 0.5 \* inv\_y[n];

114

115  if (!is\_constant\_struct<T\_y>::value) {

116  operands\_and\_partials.d\_x1[n]

117  += -(half\_nu+1.0) \* inv\_y[n] + 0.5 \* inv\_y[n] \* inv\_y[n];

118  }

119  if (!is\_constant\_struct<T\_dof>::value) {

120  operands\_and\_partials.d\_x2[n]

121  += NEG\_LOG\_TWO\_OVER\_TWO - digamma\_half\_nu\_over\_two[n]

122  - 0.5\*log\_y[n];

123  }

124  }

125  return operands\_and\_partials.to\_var(logp);

126  }

127

128  template <typename T\_y, typename T\_dof>

129  inline

130  typename return\_type<T\_y,T\_dof>::type

131  inv\_chi\_square\_log(const T\_y& y, const T\_dof& nu) {

132  return inv\_chi\_square\_log<false>(y,nu);

133  }

134

135  template <typename T\_y, typename T\_dof>

136  typename return\_type<T\_y,T\_dof>::type

137  inv\_chi\_square\_cdf(const T\_y& y, const T\_dof& nu) {

138

139  // Size checks

140  if ( !( stan::length(y) && stan::length(nu) ) ) return 1.0;

141

142  // Error checks

143  static const char\* function = "stan::prob::inv\_chi\_square\_cdf(%1%)";

144

145  using stan::math::check\_positive\_finite;

146  using stan::math::check\_not\_nan;

147  using stan::math::check\_consistent\_sizes;

148  using stan::math::check\_nonnegative;

149  using boost::math::tools::promote\_args;

150  using stan::math::value\_of;

151

152  double P(1.0);

153

154  check\_positive\_finite(function, nu, "Degrees of freedom parameter", &P);

155  check\_not\_nan(function, y, "Random variable", &P);

156  check\_nonnegative(function, y, "Random variable", &P);

157  check\_consistent\_sizes(function, y, nu,

158  "Random variable",

159  "Degrees of freedom parameter",

160  &P);

161

162  // Wrap arguments in vectors

163  VectorView<const T\_y> y\_vec(y);

164  VectorView<const T\_dof> nu\_vec(nu);

165  size\_t N = max\_size(y, nu);

166

167  agrad::OperandsAndPartials<T\_y, T\_dof> operands\_and\_partials(y, nu);

168

169  // Explicit return for extreme values

170  // The gradients are technically ill-defined, but treated as zero

171

172  for (size\_t i = 0; i < stan::length(y); i++)

173  if (value\_of(y\_vec[i]) == 0)

174  return operands\_and\_partials.to\_var(0.0);

175

176  // Compute CDF and its gradients

177  using boost::math::gamma\_p\_derivative;

178  using boost::math::gamma\_q;

179  using boost::math::tgamma;

180  using boost::math::digamma;

181

182  // Cache a few expensive function calls if nu is a parameter

183  DoubleVectorView<!is\_constant\_struct<T\_dof>::value,

184  is\_vector<T\_dof>::value> gamma\_vec(stan::length(nu));

185  DoubleVectorView<!is\_constant\_struct<T\_dof>::value,

186  is\_vector<T\_dof>::value> digamma\_vec(stan::length(nu));

187

188  if (!is\_constant\_struct<T\_dof>::value) {

189  for (size\_t i = 0; i < stan::length(nu); i++) {

190  const double nu\_dbl = value\_of(nu\_vec[i]);

191  gamma\_vec[i] = tgamma(0.5 \* nu\_dbl);

192  digamma\_vec[i] = digamma(0.5 \* nu\_dbl);

193  }

194  }

195

196  // Compute vectorized CDF and gradient

197  for (size\_t n = 0; n < N; n++) {

198

199  // Explicit results for extreme values

200  // The gradients are technically ill-defined, but treated as zero

201  if (value\_of(y\_vec[n]) == std::numeric\_limits<double>::infinity()) {

202  continue;

203  }

204

205  // Pull out values

206  const double y\_dbl = value\_of(y\_vec[n]);

207  const double y\_inv\_dbl = 1.0 / y\_dbl;

208  const double nu\_dbl = value\_of(nu\_vec[n]);

209

210  // Compute

211  const double Pn = gamma\_q(0.5 \* nu\_dbl, 0.5 \* y\_inv\_dbl);

212

213  P \*= Pn;

214

215  if (!is\_constant\_struct<T\_y>::value)

216  operands\_and\_partials.d\_x1[n]

217  += 0.5 \* y\_inv\_dbl \* y\_inv\_dbl

218  \* gamma\_p\_derivative(0.5 \* nu\_dbl, 0.5 \* y\_inv\_dbl) / Pn;

219  if (!is\_constant\_struct<T\_dof>::value)

220  operands\_and\_partials.d\_x2[n]

221  += 0.5 \* stan::math::gradRegIncGamma(0.5 \* nu\_dbl,

222  0.5 \* y\_inv\_dbl,

223  gamma\_vec[n],

224  digamma\_vec[n]) / Pn;

225  }

226

227  if (!is\_constant\_struct<T\_y>::value)

228  for (size\_t n = 0; n < stan::length(y); ++n)

229  operands\_and\_partials.d\_x1[n] \*= P;

230  if (!is\_constant\_struct<T\_dof>::value)

231  for (size\_t n = 0; n < stan::length(nu); ++n)

232  operands\_and\_partials.d\_x2[n] \*= P;

233

234  return operands\_and\_partials.to\_var(P);

235  }

236

237  template <typename T\_y, typename T\_dof>

238  typename return\_type<T\_y,T\_dof>::type

239  inv\_chi\_square\_cdf\_log(const T\_y& y, const T\_dof& nu) {

240

241  // Size checks

242  if ( !( stan::length(y) && stan::length(nu) ) ) return 0.0;

243

244  // Error checks

245  static const char\* function = "stan::prob::inv\_chi\_square\_cdf\_log(%1%)";

246

247  using stan::math::check\_positive\_finite;

248  using stan::math::check\_not\_nan;

249  using stan::math::check\_consistent\_sizes;

250  using stan::math::check\_nonnegative;

251  using boost::math::tools::promote\_args;

252  using stan::math::value\_of;

253

254  double P(0.0);

255

256  check\_positive\_finite(function, nu, "Degrees of freedom parameter", &P);

257  check\_not\_nan(function, y, "Random variable", &P);

258  check\_nonnegative(function, y, "Random variable", &P);

259  check\_consistent\_sizes(function, y, nu,

260  "Random variable",

261  "Degrees of freedom parameter", &P);

262

263  // Wrap arguments in vectors

264  VectorView<const T\_y> y\_vec(y);

265  VectorView<const T\_dof> nu\_vec(nu);

266  size\_t N = max\_size(y, nu);

267

268  agrad::OperandsAndPartials<T\_y, T\_dof> operands\_and\_partials(y, nu);

269

270  // Explicit return for extreme values

271  // The gradients are technically ill-defined, but treated as zero

272

273  for (size\_t i = 0; i < stan::length(y); i++)

274  if (value\_of(y\_vec[i]) == 0)

275  return operands\_and\_partials.to\_var(stan::math::negative\_infinity());

276

277  // Compute cdf\_log and its gradients

278  using boost::math::gamma\_p\_derivative;

279  using boost::math::gamma\_q;

280  using boost::math::tgamma;

281  using boost::math::digamma;

282

283  // Cache a few expensive function calls if nu is a parameter

284  DoubleVectorView<!is\_constant\_struct<T\_dof>::value,

285  is\_vector<T\_dof>::value> gamma\_vec(stan::length(nu));

286  DoubleVectorView<!is\_constant\_struct<T\_dof>::value,

287  is\_vector<T\_dof>::value> digamma\_vec(stan::length(nu));

288

289  if (!is\_constant\_struct<T\_dof>::value) {

290  for (size\_t i = 0; i < stan::length(nu); i++) {

291  const double nu\_dbl = value\_of(nu\_vec[i]);

292  gamma\_vec[i] = tgamma(0.5 \* nu\_dbl);

293  digamma\_vec[i] = digamma(0.5 \* nu\_dbl);

294  }

295  }

296

297  // Compute vectorized cdf\_log and gradient

298  for (size\_t n = 0; n < N; n++) {

299

300  // Explicit results for extreme values

301  // The gradients are technically ill-defined, but treated as zero

302  if (value\_of(y\_vec[n]) == std::numeric\_limits<double>::infinity()) {

303  continue;

304  }

305

306  // Pull out values

307  const double y\_dbl = value\_of(y\_vec[n]);

308  const double y\_inv\_dbl = 1.0 / y\_dbl;

309  const double nu\_dbl = value\_of(nu\_vec[n]);

310

311  // Compute

312  const double Pn = gamma\_q(0.5 \* nu\_dbl, 0.5 \* y\_inv\_dbl);

313

314  P += log(Pn);

315

316  if (!is\_constant\_struct<T\_y>::value)

317  operands\_and\_partials.d\_x1[n] += 0.5 \* y\_inv\_dbl \* y\_inv\_dbl

318  \* gamma\_p\_derivative(0.5 \* nu\_dbl, 0.5 \* y\_inv\_dbl) / Pn;

319  if (!is\_constant\_struct<T\_dof>::value)

320  operands\_and\_partials.d\_x2[n]

321  += 0.5 \* stan::math::gradRegIncGamma(0.5 \* nu\_dbl,

322  0.5 \* y\_inv\_dbl,

323  gamma\_vec[n],

324  digamma\_vec[n]) / Pn;

325  }

326

327  return operands\_and\_partials.to\_var(P);

328  }

329

330  template <typename T\_y, typename T\_dof>

331  typename return\_type<T\_y,T\_dof>::type

332  inv\_chi\_square\_ccdf\_log(const T\_y& y, const T\_dof& nu) {

333

334  // Size checks

335  if ( !( stan::length(y) && stan::length(nu) ) ) return 0.0;

336

337  // Error checks

338  static const char\* function = "stan::prob::inv\_chi\_square\_ccdf\_log(%1%)";

339

340  using stan::math::check\_positive\_finite;

341  using stan::math::check\_not\_nan;

342  using stan::math::check\_consistent\_sizes;

343  using stan::math::check\_nonnegative;

344  using boost::math::tools::promote\_args;

345  using stan::math::value\_of;

346

347  double P(0.0);

348

349  check\_positive\_finite(function, nu, "Degrees of freedom parameter", &P);

350  check\_not\_nan(function, y, "Random variable", &P);

351  check\_nonnegative(function, y, "Random variable", &P);

352  check\_consistent\_sizes(function, y, nu,

353  "Random variable",

354  "Degrees of freedom parameter", &P);

355

356  // Wrap arguments in vectors

357  VectorView<const T\_y> y\_vec(y);

358  VectorView<const T\_dof> nu\_vec(nu);

359  size\_t N = max\_size(y, nu);

360

361  agrad::OperandsAndPartials<T\_y, T\_dof> operands\_and\_partials(y, nu);

362

363  // Explicit return for extreme values

364  // The gradients are technically ill-defined, but treated as zero

365

366  for (size\_t i = 0; i < stan::length(y); i++)

367  if (value\_of(y\_vec[i]) == 0)

368  return operands\_and\_partials.to\_var(0.0);

369

370  // Compute ccdf\_log and its gradients

371  using boost::math::gamma\_p\_derivative;

372  using boost::math::gamma\_q;

373  using boost::math::tgamma;

374  using boost::math::digamma;

375

376  // Cache a few expensive function calls if nu is a parameter

377  DoubleVectorView<!is\_constant\_struct<T\_dof>::value,

378  is\_vector<T\_dof>::value> gamma\_vec(stan::length(nu));

379  DoubleVectorView<!is\_constant\_struct<T\_dof>::value,

380  is\_vector<T\_dof>::value> digamma\_vec(stan::length(nu));

381

382  if (!is\_constant\_struct<T\_dof>::value) {

383  for (size\_t i = 0; i < stan::length(nu); i++) {

384  const double nu\_dbl = value\_of(nu\_vec[i]);

385  gamma\_vec[i] = tgamma(0.5 \* nu\_dbl);

386  digamma\_vec[i] = digamma(0.5 \* nu\_dbl);

387  }

388  }

389

390  // Compute vectorized ccdf\_log and gradient

391  for (size\_t n = 0; n < N; n++) {

392

393  // Explicit results for extreme values

394  // The gradients are technically ill-defined, but treated as zero

395  if (value\_of(y\_vec[n]) == std::numeric\_limits<double>::infinity()) {

396  return operands\_and\_partials.to\_var(stan::math::negative\_infinity());

397  }

398

399  // Pull out values

400  const double y\_dbl = value\_of(y\_vec[n]);

401  const double y\_inv\_dbl = 1.0 / y\_dbl;

402  const double nu\_dbl = value\_of(nu\_vec[n]);

403

404  // Compute

405  const double Pn = 1.0 - gamma\_q(0.5 \* nu\_dbl, 0.5 \* y\_inv\_dbl);

406

407  P += log(Pn);

408

409  if (!is\_constant\_struct<T\_y>::value)

410  operands\_and\_partials.d\_x1[n] -= 0.5 \* y\_inv\_dbl \* y\_inv\_dbl

411  \* gamma\_p\_derivative(0.5 \* nu\_dbl, 0.5 \* y\_inv\_dbl) / Pn;

412  if (!is\_constant\_struct<T\_dof>::value)

413  operands\_and\_partials.d\_x2[n]

414  -= 0.5 \* stan::math::gradRegIncGamma(0.5 \* nu\_dbl,

415  0.5 \* y\_inv\_dbl,

416  gamma\_vec[n],

417  digamma\_vec[n]) / Pn;

418  }

419

420  return operands\_and\_partials.to\_var(P);

421  }

422

423  template <class RNG>

424  inline double

425  inv\_chi\_square\_rng(const double nu,

426  RNG& rng) {

427  using boost::variate\_generator;

428  using boost::random::chi\_squared\_distribution;

429

430  static const char\* function = "stan::prob::inv\_chi\_square\_rng(%1%)";

431

432  using stan::math::check\_positive\_finite;

433

434  check\_positive\_finite(function, nu, "Degrees of freedom parameter",

435  (double\*)0);

436

437  variate\_generator<RNG&, chi\_squared\_distribution<> >

438  chi\_square\_rng(rng, chi\_squared\_distribution<>(nu));

439  return 1 / chi\_square\_rng();

440  }

441

442  }

443 }

444

445 #endif

446

multiply\_log.hpp

traits.hpp

stan::agrad::tgamma

fvar< T > tgamma(const fvar< T > &x)

**Definition:** tgamma.hpp:15

stan::agrad::OperandsAndPartials::to\_var

T\_return\_type to\_var(double logp)

**Definition:** partials\_vari.hpp:138

stan::math::check\_positive\_finite

bool check\_positive\_finite(const char \*function, const T\_y &y, const char \*name, T\_result \*result)

**Definition:** check\_positive\_finite.hpp:12

stan::prob::inv\_chi\_square\_cdf

return\_type< T\_y, T\_dof >::type inv\_chi\_square\_cdf(const T\_y &y, const T\_dof &nu)

**Definition:** inv\_chi\_square.hpp:137

error\_handling.hpp

stan::math::multiply\_log

boost::math::tools::promote\_args< T\_a, T\_b >::type multiply\_log(const T\_a a, const T\_b b)

Calculated the value of the first argument times log of the second argument while behaving properly w...

**Definition:** multiply\_log.hpp:51

stan::length

size\_t length(const T &)

**Definition:** traits.hpp:159

stan::DoubleVectorView

DoubleVectorView allocates double values to be used as intermediate values.

**Definition:** traits.hpp:358

stan::prob::inv\_chi\_square\_log

return\_type< T\_y, T\_dof >::type inv\_chi\_square\_log(const T\_y &y, const T\_dof &nu)

The log of an inverse chi-squared density for y with the specified degrees of freedom parameter...

**Definition:** inv\_chi\_square.hpp:43

partials\_vari.hpp

stan::agrad::value\_of

T value\_of(const fvar< T > &v)

Return the value of the specified variable.

**Definition:** value\_of.hpp:16

constants.hpp

stan::is\_vector

**Definition:** traits.hpp:83

stan::agrad::lgamma

fvar< T > lgamma(const fvar< T > &x)

**Definition:** lgamma.hpp:15

stan::agrad::OperandsAndPartials

A variable implementation that stores operands and derivatives with respect to the variable...

**Definition:** partials\_vari.hpp:76

traits.hpp

stan::return\_type::type

boost::math::tools::promote\_args< typename scalar\_type< T1 >::type, typename scalar\_type< T2 >::type, typename scalar\_type< T3 >::type, typename scalar\_type< T4 >::type, typename scalar\_type< T5 >::type, typename scalar\_type< T6 >::type >::type type

**Definition:** traits.hpp:406

stan::is\_constant\_struct

Metaprogram to determine if a type has a base scalar type that can be assigned to type double...

**Definition:** traits.hpp:57

stan::math::value\_of

double value\_of(const T x)

Return the value of the specified scalar argument converted to a double value.

**Definition:** value\_of.hpp:24

stan::prob::include\_summand

Template metaprogram to calculate whether a summand needs to be included in a proportional (log) prob...

**Definition:** traits.hpp:35

stan::agrad::gamma\_q

fvar< T > gamma\_q(const fvar< T > &x1, const fvar< T > &x2)

**Definition:** gamma\_q.hpp:15

stan::agrad::OperandsAndPartials::d\_x2

VectorView< double \*, is\_vector< T2 >::value, is\_constant\_struct< T2 >::value > d\_x2

**Definition:** partials\_vari.hpp:83

stan::math::gradRegIncGamma

double gradRegIncGamma(double a, double z, double g, double dig, double precision=1e-6)

**Definition:** internal\_math.hpp:181

stan::math::check\_nonnegative

bool check\_nonnegative(const char \*function, const T\_y &y, const char \*name, T\_result \*result)

**Definition:** check\_nonnegative.hpp:52

stan::math::check\_consistent\_sizes

bool check\_consistent\_sizes(const char \*function, const T1 &x1, const T2 &x2, const char \*name1, const char \*name2, T\_result \*result)

**Definition:** check\_consistent\_sizes.hpp:13

stan::max\_size

size\_t max\_size(const T1 &x1, const T2 &x2)

**Definition:** traits.hpp:191

internal\_math.hpp

stan::prob::inv\_chi\_square\_ccdf\_log

return\_type< T\_y, T\_dof >::type inv\_chi\_square\_ccdf\_log(const T\_y &y, const T\_dof &nu)

**Definition:** inv\_chi\_square.hpp:332

stan::math::check\_not\_nan

bool check\_not\_nan(const char \*function, const T\_y &y, const char \*name, T\_result \*result)

Checks if the variable y is nan.

**Definition:** check\_not\_nan.hpp:56

stan::agrad::digamma

fvar< T > digamma(const fvar< T > &x)

**Definition:** digamma.hpp:16

value\_of.hpp

stan::agrad::OperandsAndPartials::d\_x1

VectorView< double \*, is\_vector< T1 >::value, is\_constant\_struct< T1 >::value > d\_x1

**Definition:** partials\_vari.hpp:82

constants.hpp

stan::prob::inv\_chi\_square\_rng

double inv\_chi\_square\_rng(const double nu, RNG &rng)

**Definition:** inv\_chi\_square.hpp:425

stan::agrad::log

fvar< T > log(const fvar< T > &x)

**Definition:** log.hpp:15

stan::VectorView

VectorView is a template metaprogram that takes its argument and allows it to be used like a vector...

**Definition:** traits.hpp:275

stan::math::negative\_infinity

double negative\_infinity()

Return negative infinity.

**Definition:** constants.hpp:123

stan::prob::inv\_chi\_square\_cdf\_log

return\_type< T\_y, T\_dof >::type inv\_chi\_square\_cdf\_log(const T\_y &y, const T\_dof &nu)

**Definition:** inv\_chi\_square.hpp:239

stan::prob::chi\_square\_rng

double chi\_square\_rng(const double nu, RNG &rng)

**Definition:** chi\_square.hpp:442


---

[ Stan Home Page ]

*© 2011–2014,
Stan Development Team.*
